# Effective automated pipeline for 3D reconstruction of synapses based on deep learning

**DOI:** 10.1186/s12859-018-2232-0

**Published:** 2018-07-13

**Authors:** Chi Xiao, Weifu Li, Hao Deng, Xi Chen, Yang Yang, Qiwei Xie, Hua Han

**Affiliations:** 1 Institute of Automation, Chinese Academy of Sciences, 95 Zhongguancun East Road, Beijing, 100190 China; 2 School of Future Technology, University of Chinese Academy of Sciences, 19 Yuquan Road, Shijingshan District, Beijing, 100049 China; 3 Faculty of Mathematics and Statistics, Hubei University, Institute of Automation, Chinese Academy of Sciences, 368 Youyi Road, Wuhan, China; 4 Faculty of Information Technology, Macau University of Science and Technology, Avenida Wai Long, Taipa, Macau, China; 5 Institute of Neuroscience, Chinese Academy of Sciences, 320 Yue Yang Road, Shanghai, 200031 China; 6 Center for Excellence in Brain Science and Intelligence Technology, Chinese Academy of Sciences, 320 Yue Yang Road, Shanghai, 200031 China; 7 Data Mining Lab, Beijing University of Technology, 100 Ping Le Yuan, Beijing, 100124 China

**Keywords:** Electron microscope, Synapse detection, Deep learning, Synapse segmentation, 3D Reconstruction of synapses

## Abstract

**Background:**

The locations and shapes of synapses are important in reconstructing connectomes and analyzing synaptic plasticity. However, current synapse detection and segmentation methods are still not adequate for accurately acquiring the synaptic connectivity, and they cannot effectively alleviate the burden of synapse validation.

**Results:**

We propose a fully automated method that relies on deep learning to realize the 3D reconstruction of synapses in electron microscopy (EM) images. The proposed method consists of three main parts: (1) training and employing the faster region convolutional neural networks (R-CNN) algorithm to detect synapses, (2) using the z-continuity of synapses to reduce false positives, and (3) combining the Dijkstra algorithm with the GrabCut algorithm to obtain the segmentation of synaptic clefts. Experimental results were validated by manual tracking, and the effectiveness of our proposed method was demonstrated. The experimental results in anisotropic and isotropic EM volumes demonstrate the effectiveness of our algorithm, and the average precision of our detection (92.8% in anisotropy, 93.5% in isotropy) and segmentation (88.6% in anisotropy, 93.0% in isotropy) suggests that our method achieves state-of-the-art results.

**Conclusions:**

Our fully automated approach contributes to the development of neuroscience, providing neurologists with a rapid approach for obtaining rich synaptic statistics.

**Electronic supplementary material:**

The online version of this article (10.1186/s12859-018-2232-0) contains supplementary material, which is available to authorized users.

## Background

A synapse is a structure that permits a neuron (or nerve cell) to pass an electrical or chemical signal to another neuron, and it has an important responsibility in the neural system. If we consider the brain network to be a map of connections, then neurons and synapses can be considered as the dots and lines, respectively, and it can be hypothesized that the synapse is one of the key factors for researching connectomes [1–3]. In addition, synaptic plasticity is associated with learning and memory. Sensory experience, motor learning and aging are found to induce alterations in presynaptic axon boutons and postsynaptic dendritic spines [4–6]. Consequently, understanding the mechanism of synaptic plasticity will be conducive to the prevention and treatment of brain diseases. To study the correlation between synaptic growth and plasticity and to reconstruct neuronal connections, it is necessary to obtain the number, location and structure of synapses in neurons.

According to the classification of synaptic nerve impulses, there are two types of synapses: chemical synapses and electrical synapses. In this study, we focus on the chemical synapse, which consists of presynaptic (axonal) membrane, postsynaptic (dendritic) membrane and a 30-60 *nm* synaptic cleft. Because of its limited resolution, optical microscopy cannot provide sufficient resolution to reveal these fine structures. Fortunately, it is now possible to more closely examine the synapse structure due to the rapid development of electron microscopy (EM). In particular, focused ion beam scanning electron microscopy (FIB-SEM) [7] can provide nearly 5*nm* imaging resolution, which is conducive to obtaining the very fine details of ultrastructural objects; however, this technique is either limited to a small section size (0.1 *mm* × 0.1 *mm*) or provides blurred imaging.

By contrast, automated tape-collecting ultramicrotome scanning electron microscopy (ATUM-SEM) [8] offers anisotropic voxels with a lower imaging resolution in the z direction (2 *nm* × 2 *nm* × 50 *nm*), but it is capable of working with large-area sections (2.5 *mm* ×6 *mm*). Moreover, ATUM-SEM does not damage any sections; thus, the preserved sections can be imaged and analyzed many times. Considering volume and resolution, this paper employs ATUM-SEM and FIB-SEM image stacks to verify the validity and feasibility of our algorithms.

Note that EM images with higher resolution will inevitably produce more data in the same volume; thus, synapse validation requires a vast amount of laborious and repetitive manual work. Consequently, an automated synapse reconstruction pipeline is essential for analyzing large volumes of brain tissue [9]. Prior works on synapse detection and segmentation investigated a range of approaches. Mishchenko et al. [10] developed a synaptic cleft recognition algorithm to detect postsynaptic densities in serial block-face scanning electron microscopy (SBEM) [11] image stacks. However, this method was effective for synapse detection only if the prior neuron segmentation was satisfactory. Navlakha et al. [12] presented an original experimental technique for selectively staining synapses, and then they utilized a semi-supervised method to train classifiers such as support vector machine (SVM), AdaBoost and random forest to identify synapses. Similarly, Jagadeesh et al. [13] presented a new method for synapse detection and localization. This method first characterized synaptic junctions as ribbons, vesicles and clefts, and then it utilized maximally stable extremal region (MSER) to design a detector to locate synapses. However, all these works [10, 12, 13] ignored the contextual information of synapses.

For the above reasons, Kreshuk et al. [14] presented a contextual approach for automated synapse detection and segmentation in FIB-SEM image stacks. This approach adopted 35 appearance features, such as magnitude of Gaussian gradient, Laplacian of Gaussian, Hessian matrix and structure tensor, and then it employed a random forest classifier to produce synapse probability maps. Nevertheless, this approach neglected the asymmetric information produced by the presynaptic and postsynaptic regions, which led to some inaccurate results. Becker et al. [15] utilized contextual information and different Gaussian kernel functions to calculate synaptic characteristics, and then they employed these features to train an AdaBoost classifier to obtain synaptic clefts in FIB-SEM image stacks. Similarly, Kreshuk et al. [16] proposed an automated approach for synapse segmentation in serial section transmission electron microscopy (ssTEM) [17] image stacks. The main idea was to classify synapses from 3D features and then segment synapses by using the Ising model and object-level features classifier. Ref. [16] did not require prior segmentation and achieved a good error rate. Sun et al. [18] focused on synapse reconstruction in anisotropic image stacks, which were acquired through ATUM-SEM; detected synapses with cascade AdaBoost; and then utilized continuity to delete false positives. Subsequently, the variational region growing [19] was adopted to segment synaptic clefts. However, the detection accuracies of Ref. [16] and Ref. [18] were not satisfactory, and the segmentation results lacked smoothness.

Deep neural networks (DNNs) have recently been widely applied in solving medical imaging detection and segmentation problems [20–23] due to their extraordinary performance. Thus, the application of DNNs to synapse detection in EM data holds great promise. Roncal et al. [24] proposed a deep learning classifier (VESICLE-CNN) to segment synapses directly from EM data without any prior knowledge of the synapse. Staffler et al. [25] presented SynEM, which focused on classifying borders between neuronal processes as synaptic or non-synaptic and relied on prior neuron segmentation. Dorkenwald et al. [26] developed the SyConn framework, which used deep learning networks and random forest classifiers to obtain the connectivity of synapses.

**Fig. 1:**
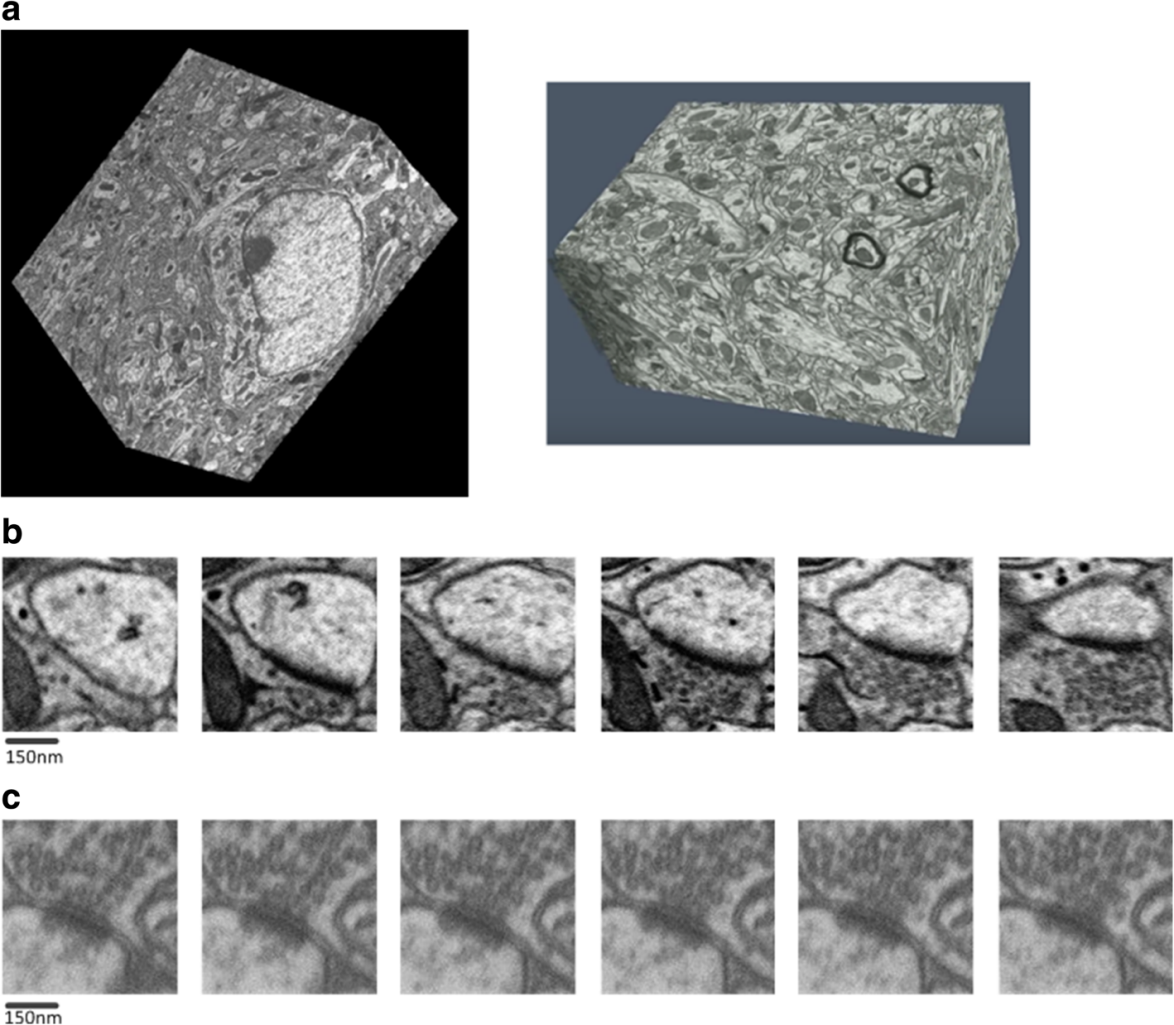
Datasets and synapses. **a** Left: An anisotropic stack of neural tissue from mouse cortex acquired by ATUM-SEM. Right: Isotropic physical sections from rat hippocampus obtained by FIB-SEM. **b** Serial synapses in ATUM-SEM images. **c** Serial synapses in FIB-SEM images. As shown, the ATUM-SEM images are the sharper ones

In this paper, we introduce a fully automated method for realizing the 3D dense reconstruction of synapses in FIB-SEM and ATUM-SEM images by combining a series of effective detection and segmentation methods. The image datasets are depicted in Fig. 1. To avoid false distinctions between a synaptic cleft and membrane, we utilize contextual information to consider the presynaptic membrane, synaptic cleft and postsynaptic membrane as a whole, and then we adopt a deep learning detector [27] to obtain the accurate localization of synapses. Subsequently, a screening method with z-continuity is proposed to improve the detection precision. To precisely segment synapses, the Dijkstra algorithm [28] is employed to obtain the optimal path of the synaptic cleft, and the GrabCut algorithm [29] is applied for further segmentation. Finally, we utilize ImageJ [30] to visualize the 3D structure of synaptic clefts, and we compare our results with other promising results obtained by Refs. [15, 18, 19, 23]. By using deep learning, z-continuity and GrabCut, our approach performs significantly better than these methods.

## Method

**Fig. 2:**
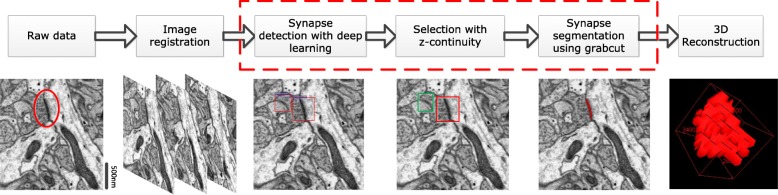
The workflow of our proposed method. Left to right: the raw data with one synapse, shown in red circles; image registration results; synapse detection results of the faster region convolutional neural networks (R-CNN); the results of screening method using z-continuity, with positive shown in red and negative in green; synaptic cleft segmentation through GrabCut; and 3D reconstruction of the synapse

The proposed automated synapse reconstruction method for EM serial sections of biological tissues can be divided into five parts, as follows: image registration (ATUM-SEM only), synapse detection with deep learning, screening method with z-continuity, synapse segmentation using GrabCut and 3D reconstruction. The related video of the 3D reconstruction is shown in Additional file 1: Video S1. In this paper, we focus on the middle three steps. Figure 2 illustrates the workflow of the proposed method.
Additional file 1: Video S1. 3D reconstruction result in Fig. 2. (MOV 946 kb)

The proposed image registration method for serial sections of biological tissue is divided into three parts: searching for correspondences between adjacent section, displacement calculations for the identified correspondences, and warping the image tiles based on the new position of these correspondences. For the correspondences searching, we adopted SIFT-flow algorithm [31], to search for correspondences between adjacent sections by extracting equally distributed grid points on the well-aligned adjacent sections. For the displacement calculation, the positions of the identified correspondences were adjusted throughout all sections by minimizing a target energy function, which consisted of the data term, the small displacement term, and the smoothness term. The data term keeps pairs of correspondences at the same positions in the x-y plane after displacement. The small displacement term constrains the correspondence displacements to minimize image deformation. The smoothness term constrains the displacement of the neighbor correspondences. For the image warping, we used the Moving Least Square (MLS) method [32] to warp each section with the obtained positions. The deformation results produced by MLS are globally smooth to retain the shape of biological specimens. The similar statement also can be seen from Ref. [33]. This image registration method not only reflects the discontinuity around wrinkle areas but also retains the smoothness in other regions, which provides a stable foundation for follow-up works.

### Synapse detection with deep learning

**Fig. 3:**
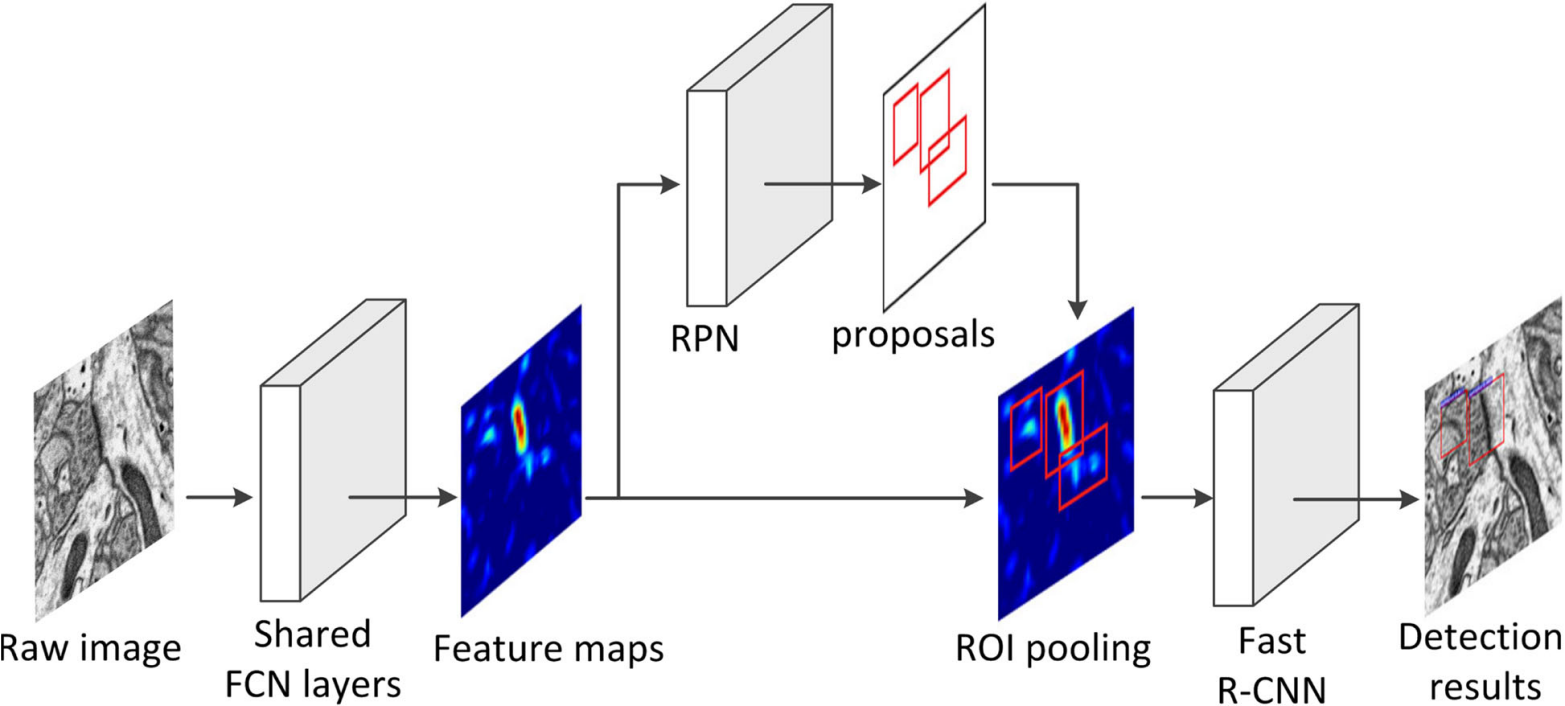
Faster R-CNN architecture. A raw image is input into a shared FCN, and then RPN is applied to generate region proposals from feature maps. Subsequently, each proposal is pooled into a fixed-size feature map, followed by the Fast R-CNN model to obtain the final detection results. This architecture is trained end-to-end with a multi-task loss

In this part, Faster R-CNN was adopted to detect synapses in EM image stacks. Faster R-CNN mainly consists of two modules: the first module is the region proposal network (RPN), which generates region proposals, and the second one is Fast R-CNN [34], which classifies the region proposals into different categories. The process of applying Faster R-CNN to detect synapses is illustrated in Fig. 3. First, we used a shared fully convolutional network (FCN) to obtain the feature maps of the raw data. The visualizations of feature maps indicate that, more neurons in the convolutional layer positively react to the visual patterns of synapses than others. Thus making it easier to recognize synapses from these maps. Subsequently, we adopted RPN to extract candidate regions from the feature maps (the architectures of shared FCN layers and RPN are illustrated in [Appendix 1]). Given the proposed regions and feature maps, the Fast R-CNN module was employed to classify the region proposals into synapse and background. In Faster R-CNN, the four basic steps of target detection, namely, region proposal, feature extraction, object classification and bounding-box regression, are unified in a deep-learning-based and end-to-end object detection system. Consequently, it is capable of guaranteeing a satisfactory result in terms of both overall detection accuracy and operation speed.

**Fig. 4:**
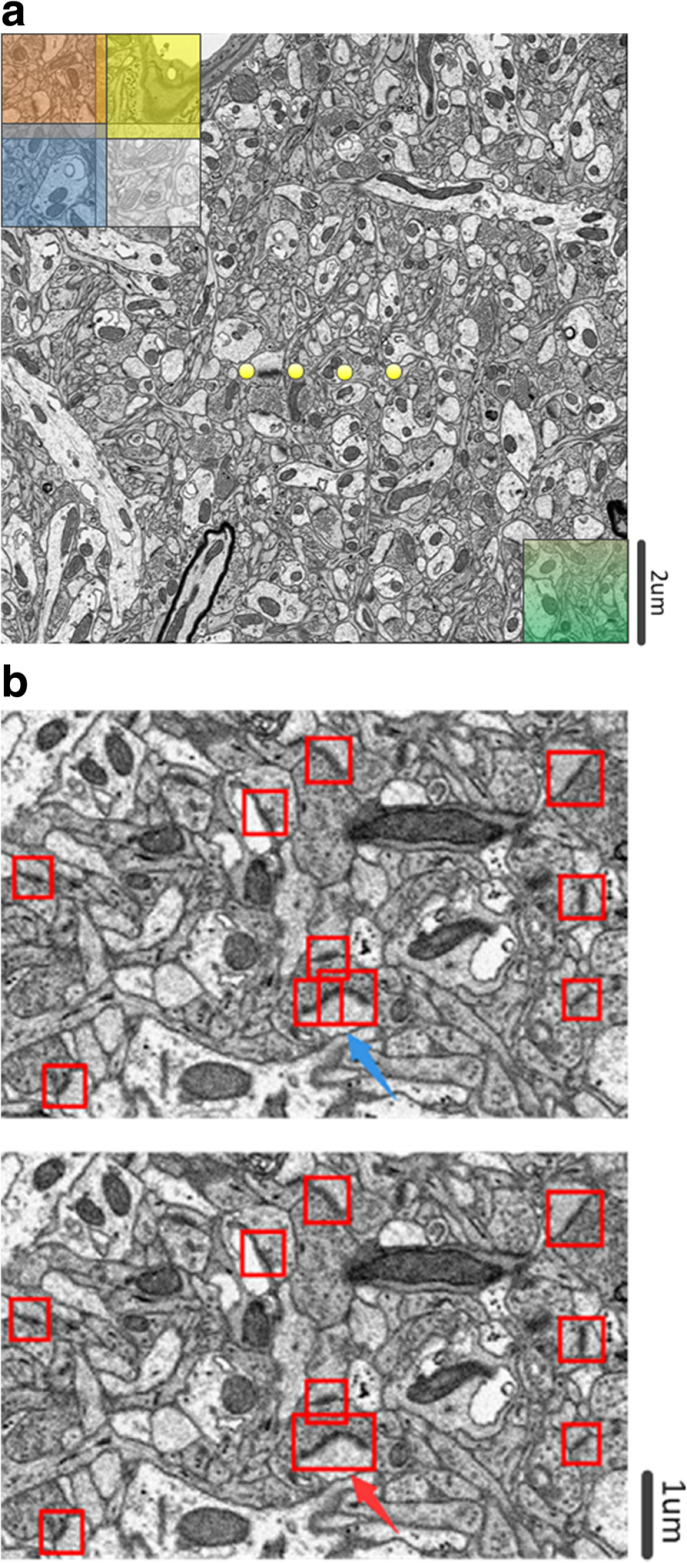
Image progressing during the use of Faster R-CNN. **a** Illustration of image clipping. **b** Top: Detection results, where the blue arrow is pointing to the duplicate detections. Bottom: Detection results with the fusion algorithm, where the red arrow is pointing to the fusion result

Faster R-CNN is widely used to train and test natural image datasets, such as PASCAL VOC and MS COCO, where the height and width ranges of these images are from 500 pixels to 800 pixels. For an EM image, its size is generally larger than that of a natural image, larger than even 8000 pixels, which requires more memory storage in the GPU. To avoid exceeding the memory of the GPU, smaller images are proposed to train Faster R-CNN. For the ATUM-SEM dataset, we divided the original ATUM-SEM images (size of 8624 ×8416) into 72 small images (size of 1000 ×1000), allowing a nearly 50 pixel overlap between each image to avoid false negatives, as shown in Fig. 4a. Similarly, we divided one original FIB-SEM image (size of 768 ×1024) into 6 overlapping small images (size of 500 ×500). In the following, the application Training Image Labeler was employed to label synapses. To avoid overfitting, we used augmentation strategy such as flip and rotation to enlarge the training dataset. Through data augmentation, the number of both training samples is greater than 7000, which is sufficient for single target detection.

The deep learning network was implemented using Caffe [35] deep learning library (The process of training the Faster R-CNN is shown in [Appendix 2]). In training process, Faster R-CNN was optimized by the stochastic gradient descend (SGD) algorithm with the following optimization hyperparameters: weight decay = 0.0005, momentum = 0.9, gamma = 0.1, learning rate = 0.0001 for numerical stability. The mini-batch size and number of anchor locations were set to 128 and 2400, respectively. In addition to ZF [36] and VGG16 [37], we also applied ResNet50 [38] as shared FCN to train Faster R-CNN. It took nearly 20-28 hours to train the network for 80000 iterations on a GeForce Titan X GPU.

Given the detection results of small images, it is easy to gather all detections and obtain the final detection results of an original image. However, synapses distribute randomly in EM images, and it is possible that one synapse coexists in two adjacent small images. In this case, this method might lead to duplicate detections, which reduces the detection precision, as illustrated in Fig. 4b. Therefore, an effective detection boxes fusion method is proposed to solve this challenge. Through observations and analyses, we find that the distributions of synapses are sparse. Suppose that there are $\mathcal {N}_{i}$ synapse detection boxes in the *i*th section, $\mathcal {S}_{i,j}$ represents the *j*th synapse detection box in the *i*th section, and $\left (c_{i,j}^{1},c_{i,j}^{2}\right)$ and $\left (c_{i,j}^{3},c_{i,j}^{4}\right)$ are the upper-left coordinates and lower-right coordinates of $\mathcal {S}_{i,j}$, respectively. If two synapse detection boxes are close enough or even overlapped, it can be concluded that these might be duplicate detections. A direct evaluation criterion for duplicate detections is the distance between synapses in the same section. The main procedure in the *i*th section is illustrated in Algorithm 1. In line 11 and 12 of Algorithm 1, $\left (c_{i,j}^{^{\prime }1},c_{i,j}^{{^{\prime }2}}\right)$ and $\left (c_{i,j}^{^{\prime }3},c_{i,j}^{^{\prime }4}\right)$ are the upper-left coordinates and lower-right coordinates of the updated $\mathcal {{S^{\prime }}}_{i,j}$, respectively.


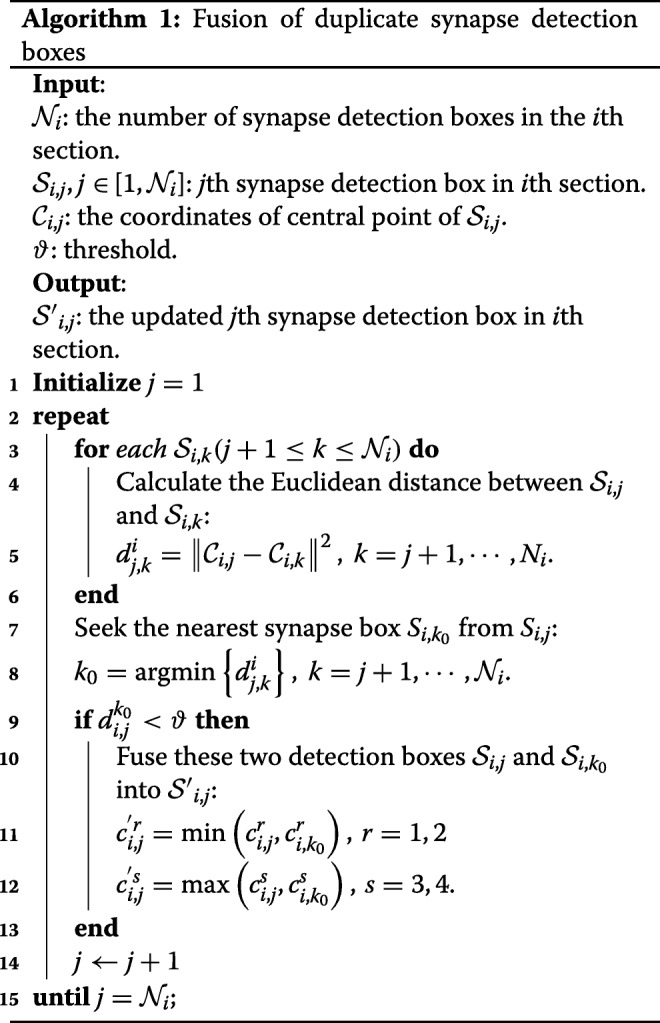


### Screening method with z-continuity

**Fig. 5:**
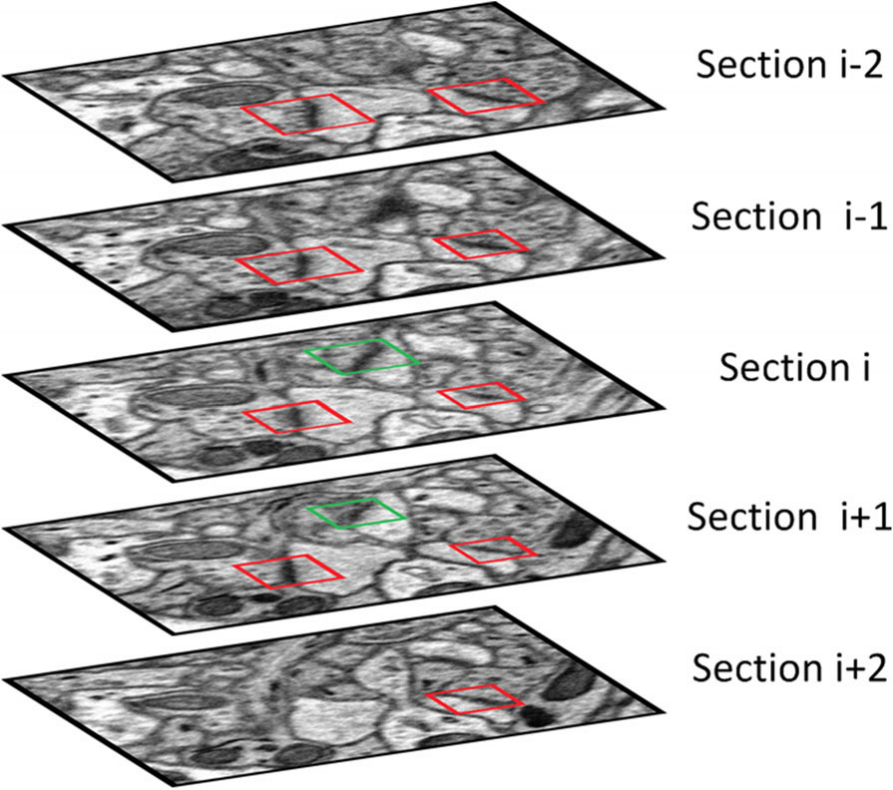
Simple schematic of screening method with z-continuity. In the case of the synapses in section *i*, we compare their location with that of the upper and lower layers; synapses that appear *L* or more times will be retained. In this figure, *L* = 3, and synapse detections in red boxes are retained while those in green boxes are removed

A synapse is a flat 3D structure with a size of nearly 400 *nm* in long axis [39], whereas the distance between adjacent section is 50 *nm* in ATUM-SEM image stacks and 5 *nm* in FIB-SEM image stacks. As shown in Fig. 5, it can be hypothesized that a real synapse is capable of appearing in several layers.

In contrast, false positives only appear in one or two layers. Therefore, we utilized z-continuity to eliminate false positives. Specifically, if a synapse detection box appears *L* times or more in the same area of continuous 2*L*−1 layers, it can be considered as a real synapse; otherwise, it is regarded as a false positive. The clear-cut principle is described in Algorithm 2.


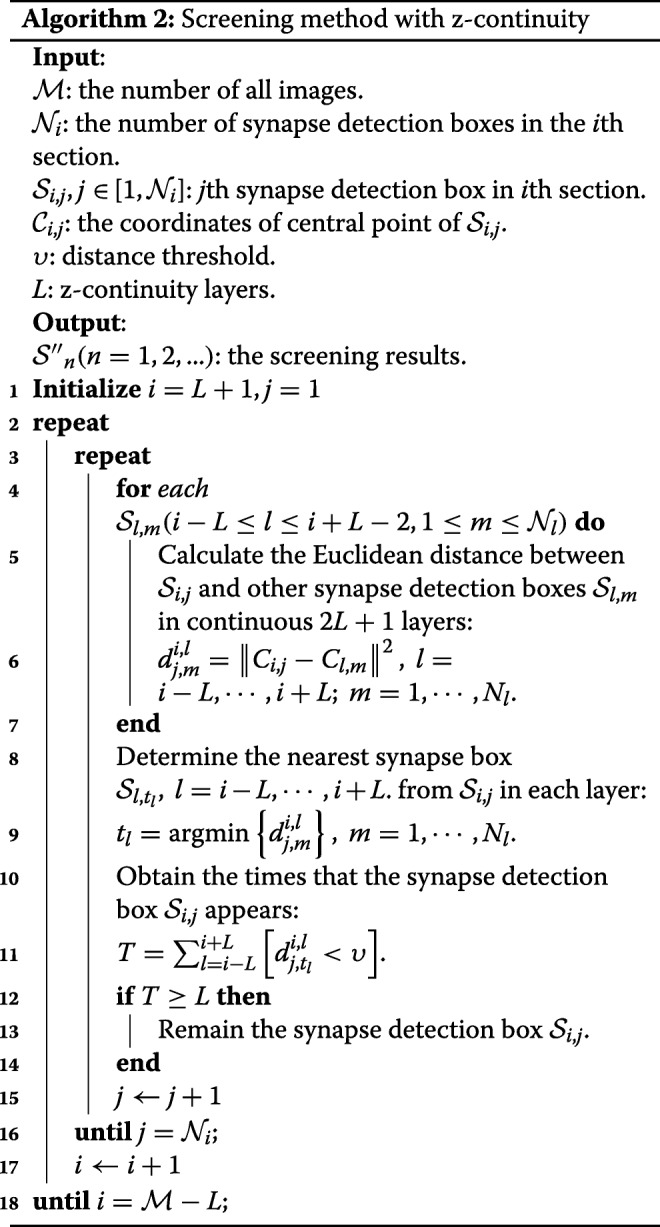


In line 11 of Algorithm 2, [·] denotes the indicator function. When T meets *T*≥*L*, we can confirm that the object detected by Faster R-CNN is a synapse with high probability; thus, this detection result $\mathcal {S}_{i,j}$ with an index in the 3D view $\mathcal {S^{\prime \prime }}_{n}(n= 1,2,\dots)$ will remain. Otherwise, it will be removed.

### Synapse segmentation using GrabCut

**Fig. 6:**
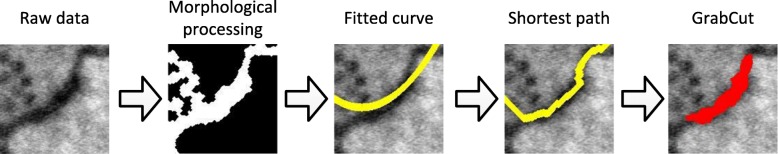
The workflow of synaptic cleft segmentation. Left to right: raw image; the result of morphological processing; fitted curve of synaptic cleft (in bold type for representation); shortest path of synaptic cleft (in bold type for representation); and segmentation result of GrabCut

Because synaptic clefts, which are at least 40 *nm* in width, are wider than other dark regions in the detection boxes, they can be segmented using several image processing methods, as illustrated in Fig. 6.

First, we converted the original detection images into binary images using an adaptive threshold. On this basis, the erode and dilate operations were employed to eliminate noise and obtain synaptic clefts. After morphological processing, synaptic clefts can be approximately located. Since most shapes of synaptic clefts are similar to quadratic curves, suitable curves are proposed to fit the structure of the synaptic clefts and obtain more refined results. We randomly selected *m* pairs of points *p*_*i*_=(*x*_*i*_,*y*_*i*_),1<*i*≤*m* from the image after morphological processing, and *m* is defined as one third of the number of white points in the corresponding image, which is empirically based. Subsequently, we employed them to fit the quadratic curve *y*=*ax*^2^+*bx*+*c*. Consequently, a series of synaptic clefts are observed as quadratic curves. Finally, we selected the starting point *p*_1_ and the ending point *p*_*n*_ from the two ends of each fitted curve, and then we calculated the shortest path [28] between *p*_1_ and *p*_*n*_.

Note that the obtained shortest path is only a curve rather than a segmentation result, and sometimes the dilated results of fitted curve and shortest path cannot effectively fit the various synaptic clefts, as shown in the Fig. 6, an effective segmentation algorithm has to be introduced. Motivated by previous researches [29], we proposed to use GrabCut algorithm for fine segmentation.

First, we considered the image as an array *α*=(*α*_1_,…,*α*_*N*_), and we regarded the segmentation result as an array *β*=(*β*_1_,…,*β*_*n*_,…,*β*_*N*_) at each pixel, *β*_*n*_={0,1} with 1 for synapse and 0 for background. The parameters *θ* denoted the distributions of synapse and background in the image. Next, we modeled a full-covariance Gaussian mixture model (GMM) [40] for synapse and background with *K* components separately. To properly use the GMM, an additional vector *k*=(*k*_1_,…,*k*_*n*_,…,*k*_*N*_) is introduced, where *k*_*n*_ is the Gaussian component corresponding to the *nth* pixel. For each pixel, the GMM component is either from the synapse model or the background model. The task of segmentation is to obtain the unknown variables $\underline {\beta }$ from the given image *α* and the model parameters $\underline {\theta }$. The Gibbs energy *E* consists of a data term $\mathcal {D}$ and smoothness term $\mathcal {S}$, which can be defined as

1 $$ E(\underline{\beta},k,\underline{\theta}, \alpha) =\mathcal{D}(\beta, k,\underline{\theta}, \alpha)+\mathcal{S}(\underline{\beta}, \alpha).  $$

The minimum of Eq. (1) can be considered as a good segmentation. In Eq. (1), the data term $\mathcal {D}$ indicates the penalty for a pixel that is classified incorrectly. According to the GMMs, it can be defined as

2 $$ \mathcal{D}(\underline{\beta}, k,\underline{\theta}, \alpha) = \sum_{n}G(\underline{\beta_{n}},k_{n},\underline{\theta},\alpha_{n}).  $$

where *G* can be expressed as

3 $$ G(\underline{\beta_{n}},k_{n},\underline{\theta},\alpha_{n}) \!= -\log p(\alpha_{n} \mid \underline{\beta_{n}},k_{n},\underline{\theta}) - \log \pi(\underline{\beta_{n}},k_{n}).  $$

In this work, *p*(·) denotes the Gaussian probability distribution, and *π*(·) represents the mixture weight.

GraphCut [41] is a one-time minimization, whereas GrabCut is an iterative minimization, and each iteration process makes the GMM parameters better for image segmentation. Initialize the trimap *T*={*T*_*S*_,*T*_*B*_,*T*_*U*_} by selecting the rectangular box. The pixels outside the box belong to background *T*_*B*_, whereas the pixels inside the box indicate “they might be synapses" and belong to *T*_*U*_, and *T*_*S*_ implies synapse. To obtain a better result, users can draw a masking area inside the box with a synapse brush and a background brush, where the pixels in different masking areas are regarded as different classes. The detailed procedures for synapse segmentation using GrabCut are described in Algorithm 3.


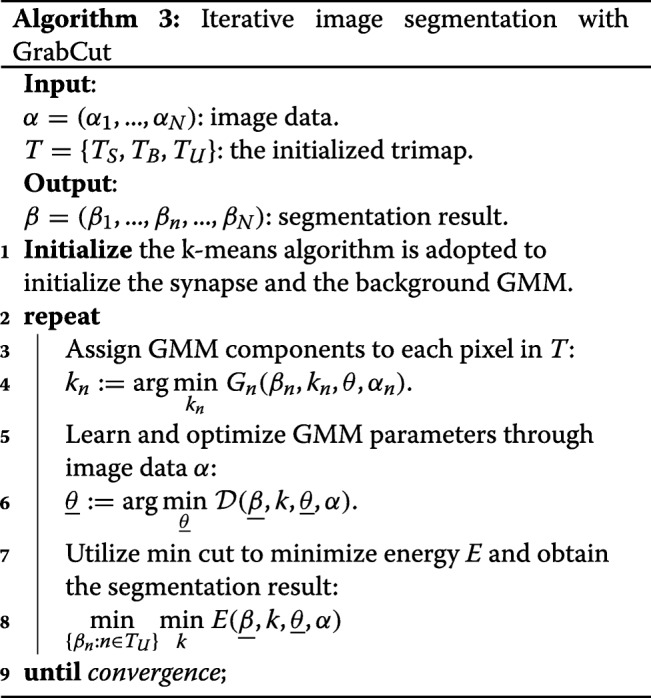


**Fig. 7:**
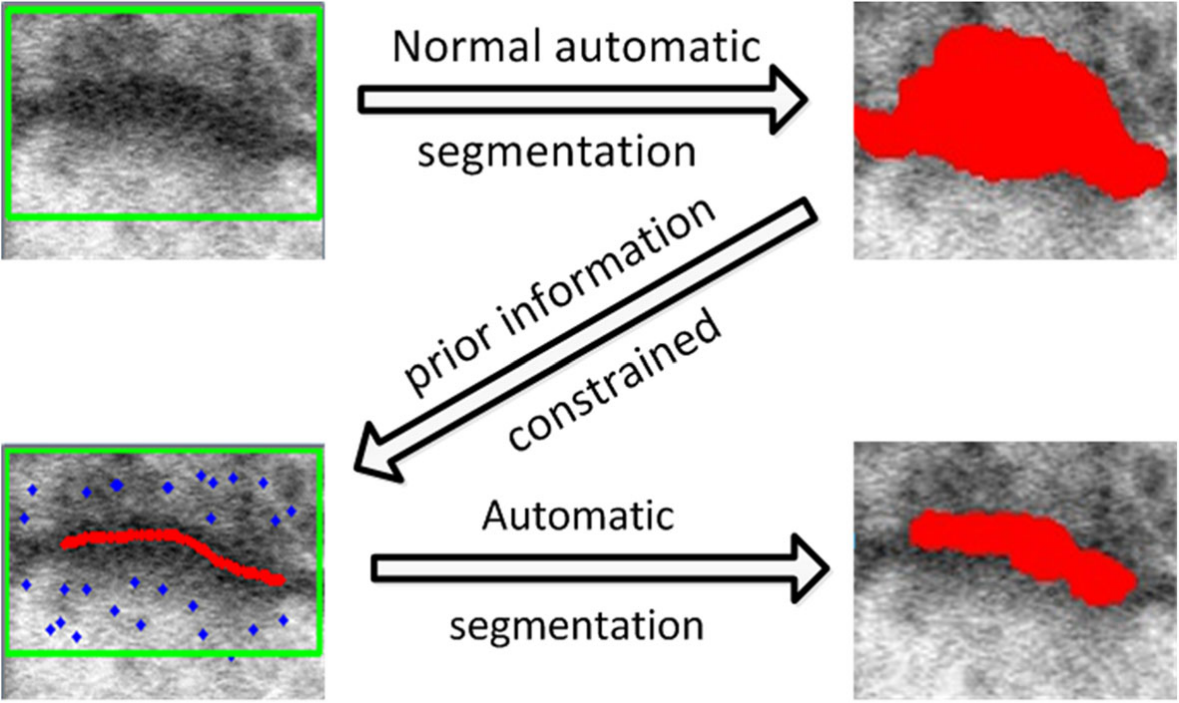
The description of synapse segmentation through GrabCut. Normal automatic segmentation methods have poor performance in EM images (top row); Therefore, further prior information is necessary. According to the existing shortest path, automatically marking with a foreground brush (red) and a background brush (blue) is sufficient to obtain a desired segmentation result (bottom row)

In Fig. 7, we present a more visual description of synapse segmentation by using GrabCut. The bounding box in green is automatically obtained by the boundary of images, which denotes the initial area *T*_*U*_ (top left corner). In the case of our datasets, the automatic segmentation result is not accurate (top right corner). For this task, we take the skeletonization of the shortest path and random sampling points as prior information, and then we apply GrabCut to synapse segmentation. The skeletonizations of the shortest path (in red) are regarded as synapse *T*_*S*_ (bottom left corner), and random sampling points (in blue) indicate background *T*_*B*_. In this case, the final segmentation result is satisfactory.

## Results and Discussion

In this section, we present several experiments on the two datasets depicted in Fig. 1a to validate our proposed method. All algorithm parameters are summarized in Table 1. Due to the difference between the two datasets, the parameters are different in the detection box fusion process and screening method with z-continuity. Parameters (subscript 1) are applied to the ATUM-SEM dataset, and parameters (subscript 2) are suitable for the FIB-SEM dataset. Although it appears that there are many parameters to tune in the pipeline, only the fusion distance threshold, z-continuity layer and z-continuity distance threshold need to be replaced when the dataset changes.

**Table 1:** Algorithm parameters and values

Parameter	Symbol	Value
Fusion distance threshold	*𝜗*_1_	100
	*𝜗*_2_	50
z-continuity layers	*L*_1_	3
	*L*_2_	20
z-continuity distance threshold	*υ*_1_	200
	*υ*_2_	100
GrabCut iterations	*T*_*G*_	10
Gaussian mixture components	*K*	5

We first present the datasets and evaluation methods. Subsequently, we adopt precision-recall curves to evaluate the performances of our detection and segmentation methods. Then, average precision (AP), F1 score and Jaccard index are employed for further quantitative analyses. Finally, we present and analyze the reconstruction results of synapses.

### Datasets and evaluation method

In this work, the specimens and ATUM-SEM sections of mouse cortex were provided by the Institute of Neuroscience, Chinese Academy of Sciences (CAS). The physical sections were imaged using an SEM (Zeiss Supra55) with an imaging voxel resolution size of 2 *nm* ×2 *nm* ×50 *nm* and dwell time of 2 *μs* by the Institute of Automation, CAS. The dataset of rat hippocampus^1^ was acquired by Graham Knott and Marco Cantoni at École Polytechnique Fédérale de Lausanne (EPFL). It is made publicly available for accelerating neuroscience research, and the resolution of each voxel is approximately 5 *nm* ×5 *nm* ×5 *nm*.

The details of the training and testing data for each dataset are summarized in Table 2. The ground truths of the datasets are annotated manually using ImageJ software. For the ATUM-SEM dataset, the training dataset contains 142 synapses in 3D view and 1522 synapses in 2D view, and the testing volume contains 723 synapses in 3D view and 7183 synapses in 2D view. For the FIB-SEM dataset, the number of synapses in training and testing are 25 and 26. Clearly, it is a time consuming and laborious process, which takes several experienced students approximately one month to obtain such a large amount of databases. respectively.

**Table 2:** Illustration of two datasets

Dataset	EM	Voxel size (*nm*^3^)	Train size	Test size
(A) Cortex	ATUM-SEM	2 ×2 ×50	8624×8416×30	7616×8576×178
(B) Hippocampus	FIB-SEM	5 ×5 ×5	1024×768×165	1024×768×165

Since our approach is composed of two primary parts, detection and segmentation, we choose different metrics for the different parts. The main metrics utilized for evaluation are as follows:

- *Precision and recall*. In this work, precision is the probability that detected synapses are correct, and recall is the probability that the true synapses are successfully detected.
  4 $$ precision = \frac{true\;positives}{true\;positives + false\;positives},  $$
  5 $$ recall = \frac{true\;positives}{true\;positives + false\;negatives}.  $$
- *Average precision*. *AP* denotes the area under the precision-recall curve, and it can be expressed as the following formula, where *P* represents precision and *R* indicates recall:
  6 $$ AP=\int_{0}^{1}P\left (R \right)dR.  $$
- *F1 score.* Since precision and recall are often contradictory, *F*1*score* is the weighted average of precision and recall, which shows the comprehensive performance of methods.
  7 $$ F1\ score = \frac{2\times P \times R}{P + R}.  $$
- *Jaccard index*. This metric is also known as the VOC score [42], which calculates the pixel-wise overlap between the ground truth (*Y*) and segmentation results (*X*).

8 $$ Jaccard~index~(X, Y) = \frac{X\bigcap Y}{X \bigcup Y}.  $$

Motivated by Ref. [43], we define that a detection or segmentation result is considered as a true positive only if the overlap between the region of the result and corresponding ground truth reaches at least 70%.

**Fig. 8:**
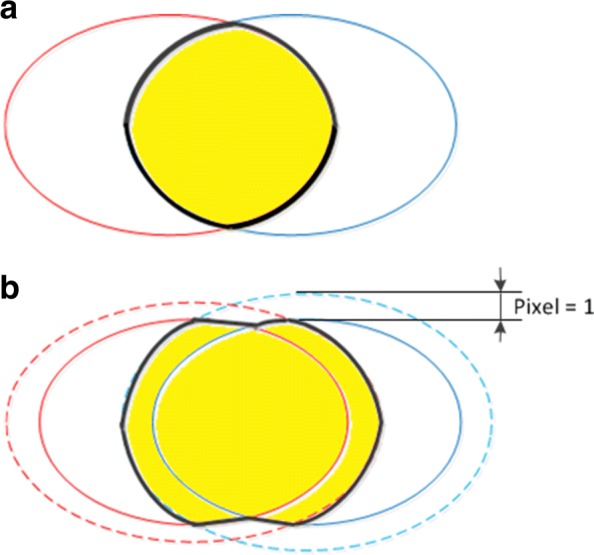
Comparison between normal overlap and 1-pixel overlap. **a** Normal overlap. **b** One-pixel overlap. Areas surrounded by red and blue solid lines denote the ground truth and segmentation result, respectively, and the yellow area represents the intersection of these two areas. In (**b**), the ground truth and segmentation result both dilate by one pixel, and it can be observed that the intersection area of (**b**) is larger than that of (**a**), which improves the fault tolerance of the evaluation

For segmentation, the shape of the synapses is always long and narrow, and the boundaries of synapses are often difficult to define. According to Ref. [15], manual annotations near synapse borders are not always accurate. Hence, due to the error in the annotations, the evaluation measure such as Jaccard index may be impacted with high probability. Inspired by the average 3-pixel error rate in Ref. [44], we define a pixel neighborhood overlap measure to eliminate this adverse effect. As depicted in Fig. 8a, the area surrounded by the red solid line denotes the ground truth (*Y*), and the area surrounded by the blue solid line indicates the segmentation result (*X*). The yellow area in Fig. 8a represents the intersection of the ground truth and segmentation result. In Fig. 8b, both the ground truth and segmentation result dilate one pixel, and the dilated ground truth (*Y*^1^) and dilated segmentation result (*X*^1^) are denoted with dashed lines. Therefore, the Jaccard index of 1-pixel overlap can be expressed as

9 $$ Jaccard~index^{1}(X, Y) = \frac{\left (X^{1}\bigcap Y \right)\bigcup \left (X\bigcap Y^{1} \right)}{X \bigcup Y}.  $$

Furthermore, we use the dilated segmentation result (*X*^1^) and dilated ground truth (*Y*^1^) to calculate the precision and recall and to obtain the 1-pixel overlap of AP. For 3-pixel overlap and 5-pixel overlap, the ground truth and segmentation result dilate three pixels and five pixels, respectively.

### Detection Accuracy

In this subsection, we evaluate the detection performance of our approach and compare it with Refs. [15, 18, 23] on different datasets in terms of precision recall curves, AP and F1 measure.

Table 3 presents the detection results of Faster R-CNN on a GeForce Titan X GPU. In Table 3, the rate shows the processing speed of different models on the test images. Through the experimental results, it can be found that the ResNet50 network provides the highest AP with an acceptance rate. Therefore, we exploited ResNet50 networks to detect synapses.

**Table 3:** Detection results of Faster R-CNN based on different models

Dataset	EM	Size	Model	AP	Rate
(A) Cortex	ATUM-SEM	1000×1000	ZF	82.0%	9 fps
			VGG16	83.2%	3 fps
			ResNet50	84.1%	4 fps
(B) Hippocampus	FIB-SEM	500×500	ZF	86.8%	36 fps
			VGG16	87.4%	12 fps
			ResNet50	90.9%	16 fps

**Fig. 9:**
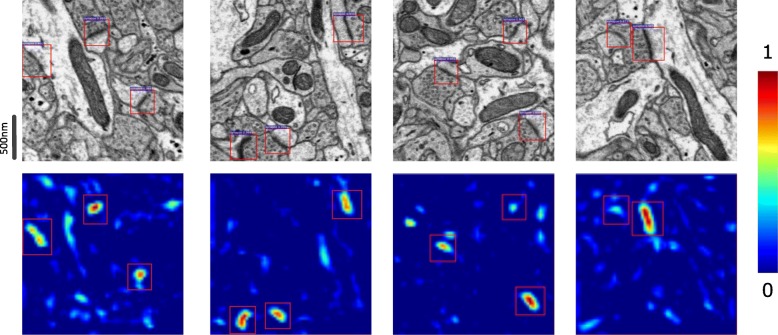
Detection results. Top: The detection results of original images. Bottom: Feature maps corresponding to the detection results

Figure 9 shows the detection results of original images and presents the feature maps extracted from the Faster R-CNN model. These figures indicate that neurons in the convolutional layer positively react to the visual patterns of synapses.

From the subsection of screening method with z-continuity, it can be hypothesized that a real synapse is capable of appearing *L* times or more in the same area of continuous 2*L*−1 layers. Therefore, we conduct several experiments by considering the cases of *L*=2, 3, 4, 5, 6, 7 on the ATUM-SEM dataset. As shown in Table 4, the detection performance (measured by AP) is highest when taking *L*=3. When *L*≥3, note that the AP decrease as the threshold *L* increase, which is mainly because of filtering the true positives.

**Table 4:** Detection performance of different threshold *L* on the ATUM-SEM dataset

Size of L	*L*=2	*L*=3	*L*=4	*L*=5	*L*=6	*L*=7
AP	77.8	86.8	83.3	75.2	70.1	66.2

**Fig. 10:**
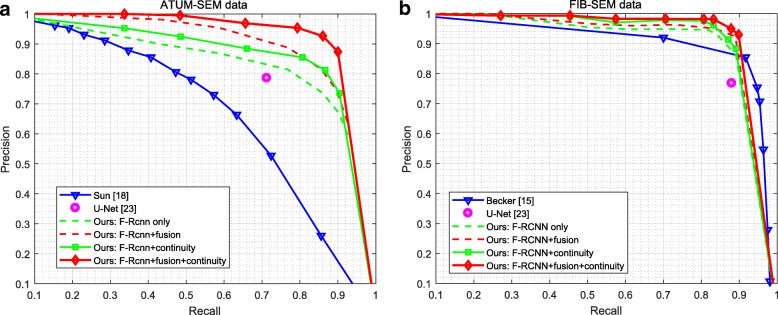
Precision-recall curves of detection for each dataset. Our approach yields better performance than that of the baseline approaches of Refs [15, 18, 23]. **a** ATUM-SEM dataset. **b** FIB-SEM dataset

Precision-recall curves are presented in Fig. 10. According to the behavior of the precision-recall plots, it can be deduced that our approach performs better than the baseline approaches of Refs. [15, 18, 23] for all recall values.

For the ATUM-SEM dataset, the performance significantly decreases since it ignores fusion and z-continuity. For the FIB-SEM dataset, the difference between synapses and other subcellular structures is significant due to its small area and simple scene. Thus, our approach achieves a higher average detection precision, and the promotion of the screening method is not enormous.

Although U-Net [23] is a simple and effective fully convolutional networks for image segmentation, which achieves good performance on different biomedical segmentation applications. Due to the imbalanced data and complex scene, the performance of U-Net is not very satisfactory in synapse segmentation task. As shown in Table 5, our approach outperforms other algorithms on different metrics. For the ATUM-SEM and FIB-SEM datasets, our approach yields 92.8 and 93.5*%* AP and 89.2 and 91.4*%* F1 score, which are higher than that of others algorithms.

**Table 5:** Quantitative detection performance for two EM datasets

**Metrics**	**Sun** [18]	**U-Net** [23]	**F-RCNN**	**F-RCNN +**	**F-RCNN +**	**F-RCNN + fusion +**
				**fusion**	**continuity**	**continuity**
**ATUM-SEM DATASET**						
AP	67.6%	74.9%	84.1%	89.7%	86.8%	92.8%
F1	64.8%	74.8%	79.3%	83.6%	83.9%	89.2%
**FIB-SEM DATASET**						
**Metrics**	**Becker** [15]	**U-Net** [23]	**F-RCNN**	**F-RCNN +**	**F-RCNN +**	**F-RCNN + fusion +**
				**fusion**	**continuity**	**continuity**
AP	90.6%	82.4%	90.9%	91.9%	92.4%	93.5%
F1	88.4%	82.0%	88.5%	90.6%	89.2%	91.4%

### Segmentation accuracy

**Fig. 11:**
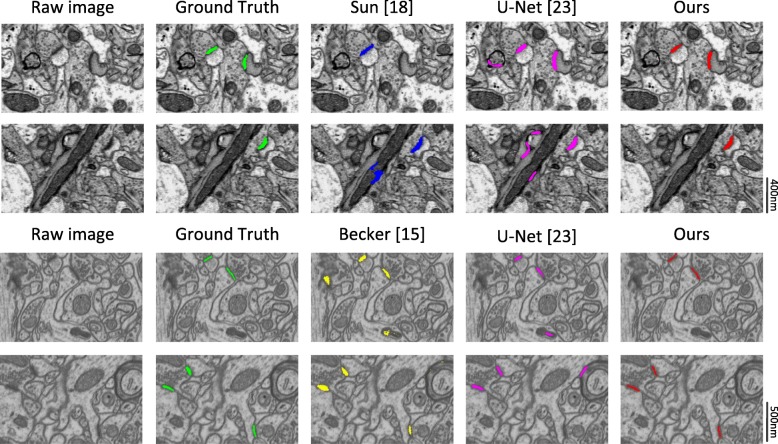
Qualitative segmentation results for two different datasets at the best threshold. Top: ATUM-SEM images, Bottom: FIB-SEM images. Note that our method achieves more accurate results as well as reducing false positives

The qualitative segmentation results are shown in Fig. 11, from which we can easily conclude that our approach achieves satisfactory results. Our approach provides more accurate results and reduces false positives, and most of the synaptic clefts are correctly segmented from the original images.

**Fig. 12:**
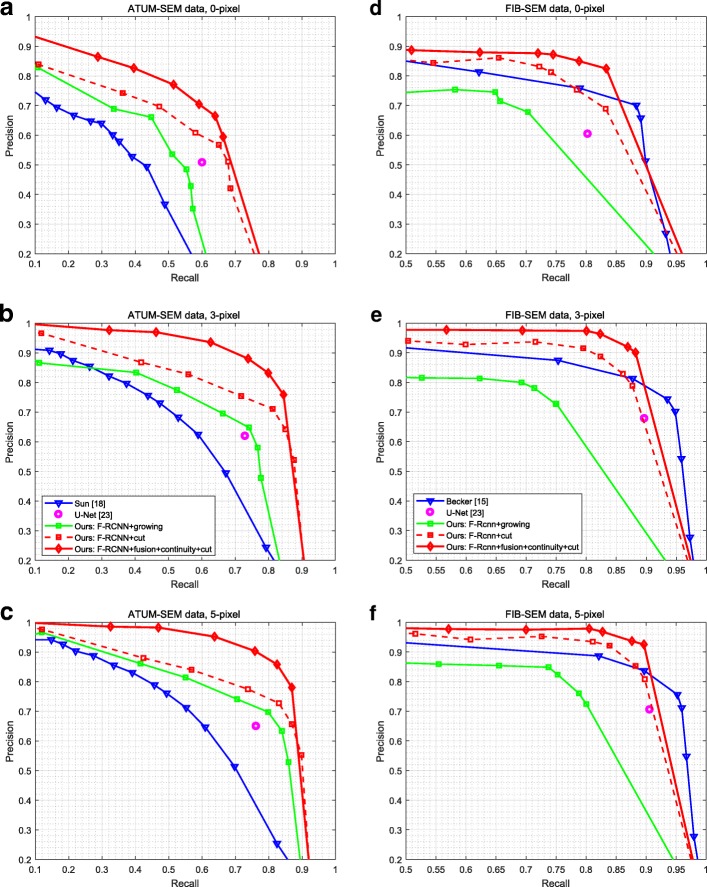
Precision-recall curves of segmentation for each dataset at different pixel overlap sizes. Cut denotes the GrabCut method, growing denotes the variational region growing method. The performance of our approach is better than that of the baseline approaches of Refs. [15, 18, 19, 23]. **a**-**c** ATUM-SEM dataset at 0, 3 and 5 pixel overlap, respectively. **d**-**f** FIB-SEM dataset at 0, 3 and 5 pixel overlap, respectively

For the quantitative analysis, evaluation of the model is performed by using precision-recall curves, AP, F1 score and Jaccard index. Precision-recall curves at different pixel overlap sizes are shown in Fig. 12, and our approach outperforms the baseline approaches of Refs. [15, 18, 19, 23] for most of the recall values and pixel overlap sizes. Note that the proposed approach with GrabCut perform much better than the approach with variational region growing [19], which verifies the effectiveness of GrabCut. In the following, we present the AP and the highest values of the F1 score and Jaccard index for all recalls at different values of pixel overlap sizes in Tables 6 and 7, which illustrate the same results. In general, our results indicate a significant improvement in the segmentation performance for each dataset.

**Table 6:** Segmentation performance for the ATUM-SEM dataset

Size of pixel	Metrics	Sun [18]	U-Net [23]	F-RCNN+	F-RCNN +	F-RCNN + fusion +
overlap				Growing [19]	GrabCut	continuity + GrabCut
0-pixel	AP	32.7%	55.4%	49.0%	57.6%	65.6%
	F1	36.3%	58.1%	53.4%	60.6%	65.2%
	Jaccard	30.6%	49.5%	43.2%	52.6%	58.1%
3-pixel	AP	56.3%	67.4%	67.8%	78.8%	86.1%
	F1	60.6%	66.9%	69.2%	75.8%	81.5%
	Jaccard	50.1%	59.0%	59.8%	69.0%	74.7%
5-pixel	AP	59.4%	70.6%	76.4%	80.3%	88.6%
	F1	62.7%	70.1%	74.4%	77.5%	84.1%
	Jaccard	53.7%	60.2%	63.5%	72.2%	76.9%

**Table 7:** Segmentation performance for the FIB-SEM dataset

Size of pixel	Metrics	Becker [15]	U-Net [23]	F-RCNN +	F-RCNN +	F-RCNN + fusion +
overlap				Growing [19]	GrabCut	continuity + GrabCut
0-pixel	AP	79.3%	70.3%	66.8%	79.4%	82.8%
	F1	78.2%	68.9%	69.4%	77.5%	82.9%
	Jaccard	61.5%	54.1%	53.5%	65.2%	70.9%
3-pixel	AP	88.0%	78.8%	74.0%	88.1%	91.6%
	F1	84.4%	77.2%	74.6%	85.4%	89.4%
	Jaccard	74.6%	60.9%	60.2%	75.7%	79.6%
5-pixel	AP	90.2%	80.6%	79.5%	90.8%	93.0%
	F1	86.6%	79.3%	78.9%	87.8%	91.1%
	Jaccard	80.3%	67.5%	67.7%	80.6%	83.7%

### 3D visualization

**Fig. 13:**
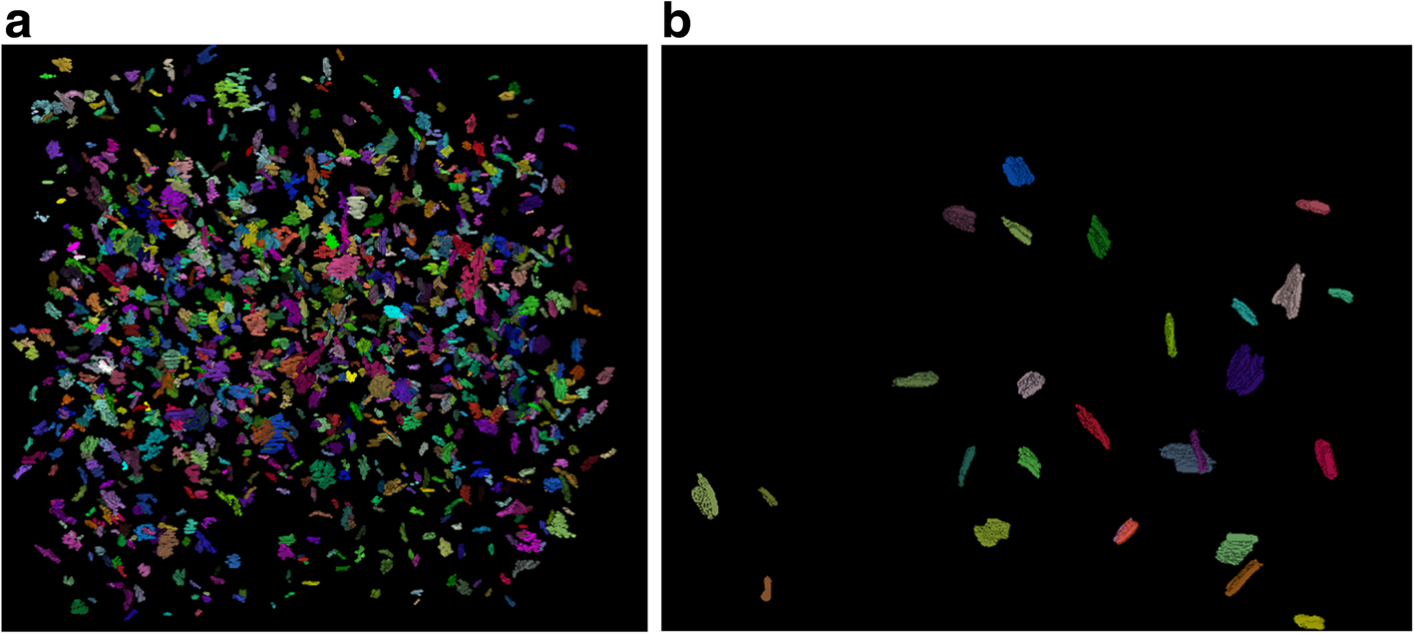
3D reconstruction of synapses. **a** Reconstruction of synapses on the ATUM-SEM dataset with a volume of 15.2 *μm*×17.2 *μm*×8.9 *μm*. **b** Reconstruction of synapses on the FIB-SEM dataset with a volume of 5.1 *μm*×3.8 *μm*×0.8 *μm*

After obtaining the segmentation results, we import them into ImageJ [30], and we show the 3D visualization of synapses for each dataset in Fig. 13. The related videos are shown in Additional file 2: Video S2 and Additional file 3: Video S3, respectively.

Additional file 2: Video S2. 3D reconstruction of synapses on the ATUM-SEM dataset. (MOV 13,499 kb)
Additional file 3: Video S3. 3D reconstruction of synapses on the FIB-SEM dataset. (MOV 2275 kb)


### Computational Efficiency

In this subsection, we illustrated the comparisons of computational efficiency in Table 8. Being different from Ref. [18] and our approach, Ref. [15] obtained the synapse segmentation results directly, thus its computational cost only contain segmentation part. For the large-scale ATUM-SEM dataset, the total detection and segmentation time of our approach is 255 mins, which is slightly slower than that of Ref. [18] (202 mins). For the FIB-SEM dataset, the test time of our approach is 8 mins, which is almost one-fourth of Ref. [15]. Note that our approach outperforms Refs. [15, 18] in precision of detection and segmentation. It can be speculated that the proposed pipeline is capable of guaranteeing a promising result in terms of both segmentation accuracy and operation speed.

**Table 8:** The comparisons of computational efficiency

Dataset	Volume (*μm*^3^)	Becker [15]	Sun [18]		Our approach	
		Segmentation	Detection	Segmentation	Detection	Segmentation
ATUM	15.2 ×17.2 ×8.9	-	48 mins	154 mins	70 mins	185 mins
FIB	5.1 ×3.8 ×0.8	30 mins	-		2 mins	6 mins

The test time was calculated on the same desktop equipped with an Intel i7-4790 CPU of 32 GB memory and a GeForce Titan X GPU

### Discussion

As mentioned in this section, our approach outperforms the approaches of Refs. [15, 18, 19, 23] on several standard metrics. However, note that the results of Ref. [15] in this paper are lower than those reported in the TMI paper. There might be two reasons for this inaccuracy. The first is that the authors of Ref. [15] offer no ground truth, and they allow us to draw it by ourselves. The second is that our performance measurements are not similar to those in Ref. [15].

Since the synapse is a flat 3D structure and the screening method with z-continuity have indeed reduced false positives in our work, which demonstrates the importance of 3D information in synapse detection problem. In addition, inspired by the promising results [45, 46], it can be speculated that the 3D network could effectively preserve and extract the 3D spatial information from volumetric data. Therefore, we believe that the extension of 2D R-CNN to 3D one could help improve the detection accuracy, and we are planning to design 3D Faster R-CNN network to detect synapses in EM data set.

## Conclusion

In this paper, we propose an effective approach to reconstruct synapses with deep learning. Our strategy is to utilize Faster R-CNN to detect the regions of synapses and then employ z-continuity to reduce false positives. Subsequently, shortest path and GrabCut are employed to obtain the synaptic clefts. Finally, we utilize our approach for the 3D reconstruction of synapses in isotropic and anisotropic datasets. The experimental results demonstrate that our algorithm enhances the precision of detection and guarantees the accuracy of segmentation, which will promote efficiency in synapse validation and benefit connectomics and synaptic plasticity analysis. Furthermore, we apply our approach to neuroscience experiments. Our automated approach helps neurologists quickly identify the number of synapses and multi-synapses in different experimental specimens, and further analyses reveal a correlation between spine formation and responses of fear-conditioned animals [4].

**Fig. 14:**
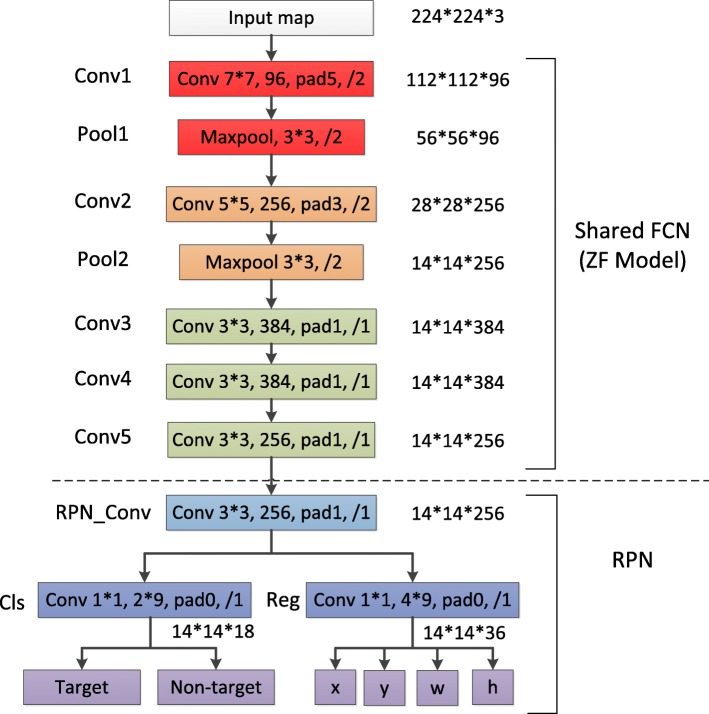
The architecture of the shared FCN layers and RPN

Despite the promising segmentation results of our approach, the segmentation process is somewhat tedious, and the efficiency and accuracy of traditional segmentation algorithms can be increased. For this task, future work will focus on detection and segmentation using end-to-end 3D deep neural networks, which will enhance both speed and accuracy for synapse reconstruction algorithm.

## Appendix 1: The architecture of shared FCN layers and RPN

Faster R-CNN mainly consists of two modules, the first module is RPN that generates region proposals, the second one is Fast R-CNN [34] which classifies the region proposals into different categories. In practice, the architecture of RPN and Fast R-CNN are fixed, ZF [36] and VGG16 [37] are applied as shared FCN to train the whole network. Here we introduce the shared FCN and RPN modules in details.

**Fig. 15:**
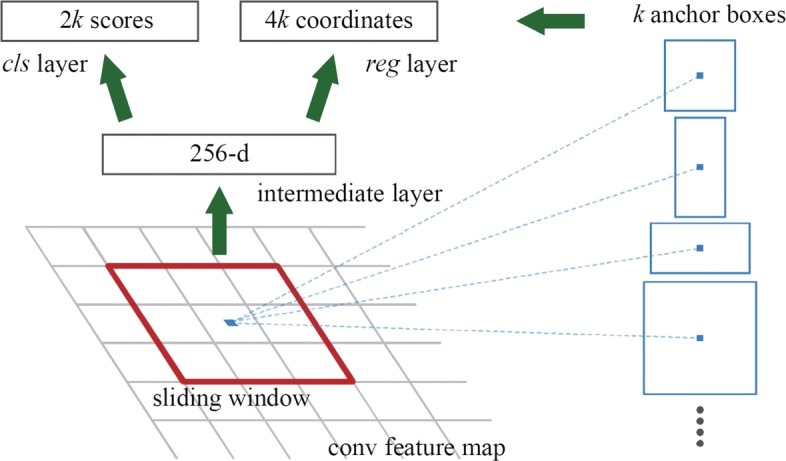
The schematic of RPN (credited by [27])

The architecture of the shared FCN layers (eg. ZF) and RPN is showed in Fig. 14. The structure above the dotted line is ZF network, and the structure below the dotted line is RPN network. Assuming the size of the input map is 224 ×224 ×3. The parameters of Conv1 layer are as follows: the kernel size is 7 ×7, the channels of feature maps is 96, the padding size and the stride size is 5 and 2, respectively. In ZF, each convolution layer is followed by batch normalization and rectified linear unit (RELU), and the size of output feature maps is 14 ×14 ×256. In RPN, it first takes a 3×3 convolutional kernel (sliding window) in the input feature maps. In the center of sliding window (illustrated in Fig. 15), there are *k* (*k*=9) anchor boxes with different scales (128, 256, 512) and aspect ratios (1 :1, 1 :2, 2 :1), which are used to solve the multi-scale target tasks in detection. In what follows, *cls* layer and *reg* layer are employed for classification and border regression respectively. *cls* layer generates two elements to determine the probability of candidates, while the *reg* layer produces four coordinate elements (*x*,*y*,*w*,*h*) to identify the location of candidates. Finally, according to the probability of candidates, RPN selects the top 300 region proposals as the input of Fast R-CNN for further classification.

## Appendix 2: The process of training the Faster R-CNN

Since RPN and Fast R-CNN share the same feature extraction network, we utilized the alternating training method to train the network with features shared, the training process is described as follows:

**Step 1:** Initialize RPN network parameters by using ImageNet pre-trained model, and then fine-tune RPN network.

**Step 2:** Utilize the region proposals generated by the step-1 RPN to train Fast R-CNN detection network. In this process, ImageNet pre-training model is also used to initialize Fast R-CNN network parameters.

**Step 3:** Keep the shared FCN layers to be fixed. Subsequently, apply step-2 Fast R-CNN network and train data to re-initialize RPN, and only fine-tune the layers unique to RPN.

**Step 4:** Employ step-3 RPN and train data to fine-tune Fast R-CNN with the shared FCN layers fixed.

The above four steps enable RPN and Fast R-CNN networks to share the convolutional layers and form a unified Faster R-CNN network.

## Abbreviations

ATUM-SEM: Automated tape-collecting ultramicrotome scanning electron microscopy
AP: Average precision
CAS: Chinese Academy of Sciences
DNNs: Deep neural networks
EM: electron microscopy
EPFL: É
cole Polytechnique Fédérale de Lausanne FCN: Fully convolutional network
FIB-SEM: Focused ion beam scanning electron microscopy
GMM: Gaussian mixture model
MLS: Moving least squares
MSER: Maximally stable extremal region
R-CNN: Region convolutional neural networks
RPN: Region proposal network
SBEM: Serial block-face scanning electron microscopy
SIFT: Scale-invariant feature transform
ssTEM: Serial section transmission electron microscopy
SVM: Support vector machine
TMI: Transactions on medical imaging

## Footnotes

1
http://cvlab.epfl.ch/data/em
